# Protocol for the isolation and characterization of extracellular vesicles from human mesenchymal stromal cells

**DOI:** 10.1016/j.xpro.2026.104745

**Published:** 2026-08-11

**Authors:** Abigail Collins, Jennifer Liddle, Jeremy Balsbaugh, Joanne Walker, Heather Wanczyk, Christine Finck, Hala Saneh

**Affiliations:** 1 Department of Pediatrics, University of Connecticut, Farmington, CT 06030, USA; 2 Proteomics and Metabolomics Facility, Center for Open Research Resources & Equipment, University of Connecticut, Storrs, CT 06269, USA; 3 Department of Pediatric Surgery, Connecticut Children’s Medical Center, Hartford, CT 06106, USA; 4 Department of Neonatology, Connecticut Children’s Medical Center, Hartford, CT 06106, USA

**Keywords:** Cell culture, Flow Cytometry, Protein expression and purification, Stem Cells

## Abstract

Here, we present a protocol for isolating extracellular vesicles (EVs) from the conditioned medium of human mesenchymal stromal cells (MSCs) and characterizing them using multiple modalities. We describe steps for enriching EVs by ultrafiltration and size-exclusion chromatography. We then detail procedures for imaging flow cytometry, transmission electron microscopy, nanoparticle tracking analysis, and the extraction of EV protein and RNA content. This workflow enables an integrated assessment of MSC-derived EVs to inform their use in diagnostic or therapeutic applications.

## Highlights

• Culture MSCs under serum-free conditions and collect conditioned media
• Isolate EVs by ultrafiltration followed by size-exclusion chromatography
• Characterize EVs by multimodal analysis, including protein and RNA profiling

## Abstract (editor highlights)

Publisher’s note: Undertaking any experimental protocol requires adherence to local institutional guidelines for laboratory safety and ethics.

## Graphical abstract

**Figure:**
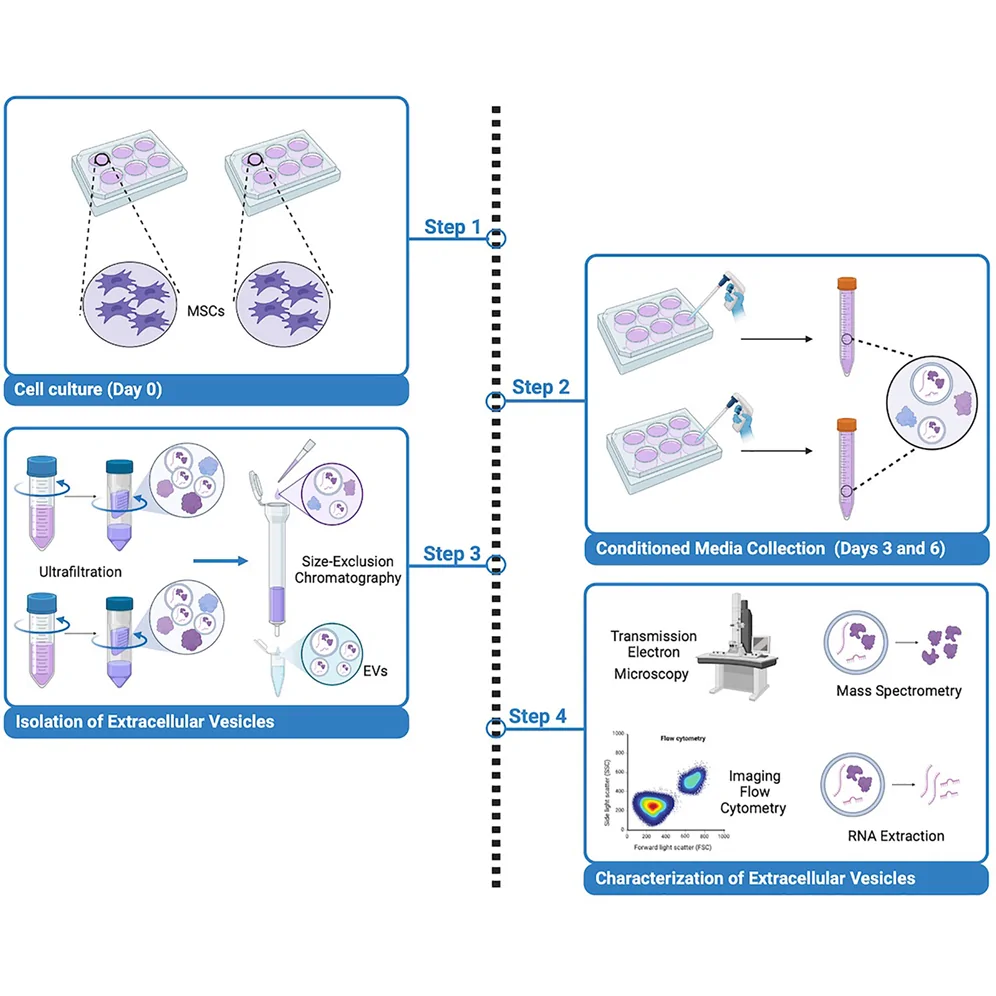

## Before you begin

The protocol below describes the procedures we use to isolate and characterize EVs from MSCs in alignment with the International Society for Extracellular Vesicles (ISEV) “Minimal Information” (MISEV) guidelines. While this workflow can be adapted to other cell types, key parameters, including culture conditions, initial cell number, seeding density, and volume of conditioned medium, must be carefully optimized for each cell source.

The choice of EV isolation method should be guided by the intended downstream application. In our experience, size-exclusion chromatography (SEC) preserves EV integrity, making it suitable for functional studies. When EVs are isolated for diagnostic or other purposes, alternative approaches may be appropriate. There is no single “universal” or “optimal” isolation technique: some strategies maximize yield at the cost of specificity, while others prioritize purity or subtype specificity. Therefore, investigators should select the isolation technique that best aligns with their experimental goals, carefully balancing yield, purity, and functionality. Transparent and thorough reporting of the chosen methods is essential to ensure reproducibility and adherence to current EV-research standards.

### Innovation

This protocol provides an integrated end-to-end workflow for the isolation and comprehensive characterization of MSC-derived EVs. It uniquely combines, within a single coherent pipeline, EV purification with multimodal characterization, including imaging flow cytometry, transmission electron microscopy, nanoparticle tracking analysis, and protein and RNA extraction for downstream omics analyses. All steps are specifically optimized for EVs derived from MSCs cultured under serum-free xeno-free conditions. We thoroughly report our experience with cell seeding density, conditioned media handling, ultrafiltration limits, SEC fraction selection, and EV input requirements for molecular analyses. Methods are refined and integrated into a unified pipeline that balances EV integrity, purity, and analytical depth. By enabling reproducible structural, phenotypic, and molecular validation of MSC-EVs, this workflow supports rigorous application-driven EV research and facilitates transparent comparison across EV studies.

### Institutional permissions

MSCs used to establish this protocol were derived from either bone marrow (BM-MSCs) or adipose tissue (AD-MSCs). Both established cell lines and patient-derived cells were utilized. Patient-derived MSCs used in this study were obtained under an Institutional Review Board (IRB)-approved protocol (CCMC IRB# 17-188) with donor consent and in accordance with relevant ethical guidelines and regulatory standards. Researchers intending to adapt or use this protocol should obtain appropriate institutional approvals prior to initiating experiments involving human cells or tissues, in accordance with local, national, and institutional regulations.

## Key resources table

**Table:** 

REAGENT or RESOURCE	SOURCE	IDENTIFIER
**Antibodies**		

Anti-Human CD63, APC (1:40)	Exbio	1A-343-T100
Anti-Human CD9, APC (1:40)	Exbio	1A-208-T100
Anti-Human CD81, APC (1:40)	Exbio	1A-558-T100

**Biological samples**		

AD-MSCs	Patients	IRB # 17-188

**Chemicals, peptides, and recombinant proteins**		

CTS TrypLE Select enzyme	Gibco	A1285901
Sodium Hydroxide (NaOH)	Spectrum Chemical	S-224
Carboxyfluorescein Succinimidyl Ester (CFSE)	Invitrogen eBioscience	65-0850-84
1% Uranyl Acetate	Electron Microscopy Sciences	NC1375332
2,2,2-trifluoroethanol (TFE)	Thermo Scientific	A10788.22
Molecular Grade Water, Optima LC/MS	Fisher Chemical	W6-1
Ammonium bicarbonate (powder/certified)	Fisher Chemical	A643500
DL-1,4-dithiothreitol (DTT)	Thermo Scientific	16568–0250
Iodoacetamide	Thermo Scientific	122275000
Porcine modified sequencing grade trypsin (protease)	Promega	V5113
Formic acid, Optima LC/MS Grade	Fisher Chemical	A117-50
Acetonitrile, Optima LC/MS	Fisher Chemical	A955-1

**Critical commercial assays**		

qEV1/70 nm	iZon	IC1-70
ExoRNeasy Midi/Maxi kit	Qiagen	77023
HighSensitivity RNA ScreenTape	Agilent TapeStation	5067-5579/5580/5581

**Experimental models: Cell lines**		

BM-MSCs Passage 2–4	ATCC	Cell line PCS-500-012

**Software and algorithms**		

Cytek IDEAS image analysis	Cytek	User guide: https://cytekbio.com/blogs/resources/ideas-image-data-exploration-and-analysis-software-users-guide
AMT image capture engine	AMT	User guide: https://0201.nccdn.net/4_2/000/000/038/2d3/AMT-Camera-Manual.pdf
MaxQuant software (v2.0.2.0)	Cox Lab	Download: https://cox-labs.github.io/coxdocs/ User guide: https://cox-labs.github.io/coxdocs/MQ_FirstSteps.html
Scaffold (v5)	Proteome Software	User guide: https://cdn.proteomesoftware.net/user_guides/scaffold_5_user_guide.pdf
msDialogue	Xiao S, Moore T, Watt C (2025)	msDiaLogue: Analysis and Visuals for Data-Independent Acquisition Mass Spectrometry Data. R package version0.0.6, https://github.com/uconn-scs/msDiaLogue

**Other**		

StemPro MSC SFM XenoFree media kit	Gibco	A1067501
CELLstart substrate	Gibco	A1014201
GlutaMAX CTS TM 100×	Gibco	1286001
Gentamicin 10 mg/mL	Gibco	15710064
PBS, pH 7.4	Gibco	10010023
Automatic Fraction Collector	iZon	AFC-V2
Amicon Ultra-15 centrifugal filters	MilliporeSigma	UFC905024
Pierce C18 Spin Columns (desalting columns)	Thermo Scientific	89870
SureSTART 0.3 mL Glass Screw Top Microvials (injection vials)	Thermo Scientific	6PSV9-03FIVAP

## Step-by-step method details

### Cell culture and collection of conditioned media

**Timing: 6 days**

1. Prepare StemPro MSC complete serum-free xeno-free medium (Table 1).
  a. In a sterile 50 mL conical tube, combine 49 mL StemPro MSC basal medium, 0.5 mL StemPro MSC supplement, 0.5 mL Glutamax (100×), and 50 μL Gentamicin (final concentration of 10 μg/mL) (Table 2). Mix gently.
  b. Store the prepared StemPro MSC complete medium at 4°C until use. Pre-warm the medium to 37°C prior to cell seeding.

***Note:*** Complete medium remains stable for up to 2 weeks when stored at 4°C.

2. Coat a 6-well culture plate with CELLstart substrate.
  a. Dilute CELLstart substrate 1:50 in Dulbecco’s Phosphate Buffered Saline (DPBS) with Ca/Mg and pipette gently to mix.
  b. Add 750 μL of diluted CELLstart substrate per well to the 6-well plate.
  c. Incubate at 37°C, 5% CO2 for 1 to 2 h before cell seeding. Alternatively, if the plate is coated the day before use, store it at 4°C, sealed with paraffin to prevent drying.
3. MSC seeding and conditioned media (CM) collection.
  a. Remove CELLstart solution from the plate immediately before cell seeding.
  b. Seed the 6-well plate with a total of 0.5 × 10^6^ cells, targeting 2 mL of media per well.
  c. Collect CM over two consecutive 72-h periods: Day 3 and Day 6 of culture. Combine media from all six wells (2 mL/well) to yield approximately 12 mL CM per collection period.
    ***Note:*** At Day 3, wash cells with phosphate buffer solution (PBS) before replenishing with fresh complete medium.
  d. Store CM at −80°C until EV isolation or proceed with EV isolation on the day of collection. If CM has been previously frozen, thaw it overnight at 4°C prior to use.
    ***Note:*** In our practice, EVs are isolated within 2 weeks of CM collection.

### EV isolation

**Timing: 2–3 h**

Multiple separation techniques exist for isolating EVs from conditioned media (differential centrifugation, density-gradient centrifugation, precipitation, immunomagnetic capture, flow-based separation techniques, size-exclusion chromatography, etc.). The choice of the method should balance recovery (yield) with specificity (purity) based on the objectives of the isolation and downstream analyses.^1^^,^^2^^,^^3^^,^^4^ In our workflow, we use ultrafiltration (UF) followed by size-exclusion chromatography (SEC) to isolate EVs from MSCs-conditioned media.

4. Low Speed Centrifugation Steps.
  a. Centrifuge each batch of collected conditioned media (12 mL volume) at 500 × *g* for 10 minutes at room temperature to pellet and remove residual cells and large cellular debris. Collect supernatant.
  b. Centrifuge the collected supernatant at 10,000 × *g* for 10 minutes at 4°C to remove larger microvesicles and apoptotic bodies. Collect supernatant.

**Figure 1:**
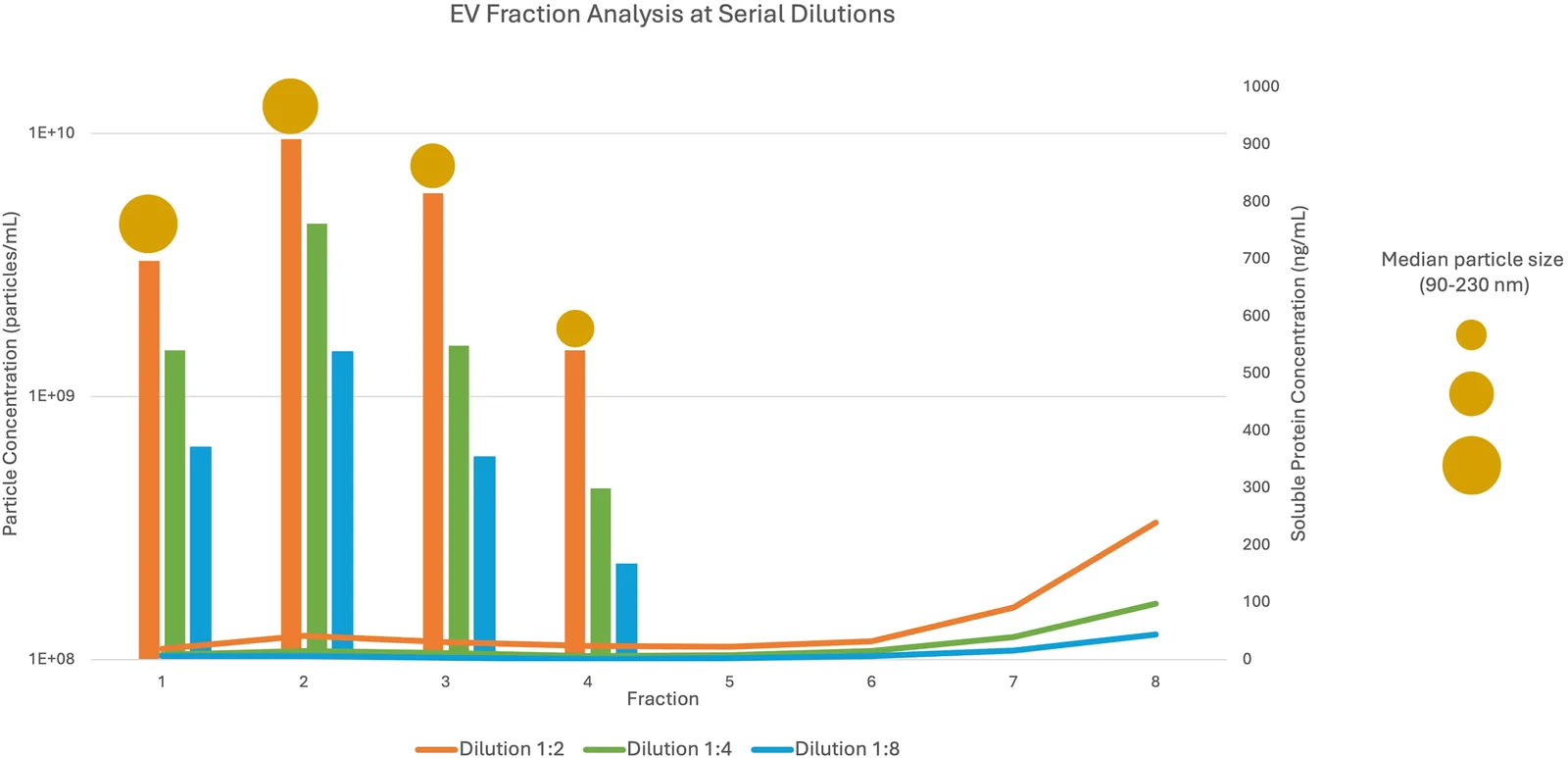
EV fraction analysis across serial dilutions EV samples were serially diluted (1:2, 1:4, 1:8) prior to SEC to assess reproducibility. Particle concentration and size distribution were measured by nanoparticle tracking analysis, and soluble protein contamination was quantified using BCA assay. Across dilutions, EV yield and size distribution were consistent. Protein contamination increased in later fractions. Fraction F3 showed optimal enrichment of small EVs (<200 nm) with minimal protein contamination and was selected for downstream analyses.
5. Ultrafiltration and Concentration.
  a. Load the supernatant (about 12 mL) into a pre-rinsed 50 kDa molecular weight cutoff ultrafilter of an Amicon 15 mL centrifugal unit.
    ***Note:*** Pre-rinse Amicon ultracentrifugal filters with sterile PBS prior to sample loading to remove residual glycerin and membrane preservatives. Do not allow the membrane to dry out once wet. If the ultrafilter is not used immediately after rinsing, leave some sterile PBS over the membrane until the device is used.
  b. Centrifuge at 3,260 × *g* at room temperature for about 6 minutes until a final volume of approximately 1 mL is obtained. Adjust centrifugation time as needed to reach the target volume. If the volume obtained is below 1 mL, add sterile filtered PBS to bring the sample to 1 mL.
    **CRITICAL:** To minimize contamination, use PBS that is sterile and filtered through 0.1 μm pore-size filters.
    **CRITICAL:** Do not concentrate multiple conditioned media preparations into a single 1 mL volume using the same Amicon centrifugal filter unit. This is a limiting step in the workflow. A maximum of 15 mL of media can be processed per filter unit in total. Do not reload or reuse the same filter to process additional volume. If larger volumes are required, perform the concentration step in separate centrifugal filter units and proceed with EV isolation for each preparation independently.
  c. Immediately recover the concentrated sample from the filter unit and transfer to a microcentrifuge tube.
  d. Centrifuge the concentrate at 10,000 × *g* for 10 minutes at 4°C to pellet precipitates and protein aggregates.
  e. Without disturbing the pellet, transfer the supernatant to a fresh tube for subsequent loading onto the qEV size-exclusion chromatography column.
6. Size Exclusion Chromatography.
  a. In a sterile hood, set up the iZONs Automated Fraction Collector (AFC) by installing the nozzle, aligning the carousel, and confirming proper placement of the waste tube.
  b. The iZon AFC screen will provide prompts for calibration. Follow these as directed, then attach the qEV1 mount and qEV1 70 nm size-exclusion chromatography column.
  c. Set the run parameters as follows:
    i. Fraction count: 3 (we will refer to these fractions as F1, F2, and F3).
    ii. Purified Collection Volume (PCV): 700 μL (this is the volume of each fraction).
    iii. Buffer Volume (BV): 4 mL (also called the void volume; this is the initial volume discarded by the collector before fraction collection begins).
      Flow through the column is gravity driven, and the AFC operates at room temperature.
  d. Before starting the collection, turn the blower off to minimize vibration of the surface supporting the AFC and ensure accurate fraction volumes during collection. Maintain aseptic handling by strictly following standard sterile techniques and minimizing exposure to the environment.
  e. Select ‘Start Collection’ and follow prompts on the screen. Attach the buffer reservoir to the top of the column and add 27 mL of sterile filtered PBS to flush the column.
  f. After the flush is complete, load the sample onto the empty column. Once the AFC indicates that the sample has fully entered the column, add 8 mL of sterile filtered PBS. The void volume is discarded first, and the fractions automatically collected.
    ***Note:*** Always use PBS as the solvent.
  g. Flush the column with PBS between samples. Clean with 0.5M NaOH between different sample types or sources and when the purification is complete.
    **CRITICAL:** Do not allow the column to dry. During the procedure, load the sample immediately after the flush.
    ***Note:*** Columns may be reused up to 5 times and should be stored at 4°C after use. During storage, ensure that some PBS remains in the column.
    **CRITICAL:** After the final run of the day and if the nozzle will be later reused, spray it thoroughly with 70% ethanol to prevent bacterial growth and contamination, and store it in a sterile tube or container.
  h. Retain fraction F3 from each sample and store at − 80°C for downstream characterization or other applications. Avoid repeated freeze-thaw cycles; if multiple downstream applications are anticipated, aliquot samples prior to storage.
    ***Note:*** We have performed a prior validation step assessing EV fractions yield and purity using serial dilutions (1:2, 1:4, and 1:8) of samples prior to loading onto the column (Figure 1). Total particle yield and size distribution were quantified across fractions via Nanoparticle Tracking Analysis, and co-isolated soluble protein was assessed using the Bicinchoninic Acid (BCA) assay. Early fractions (F1-F3) were found to be enriched in nanoparticles that are larger than 70 nm (as expected based on the column specifications), whereas later fractions showed increasing co-isolation of soluble proteins. A progressive decrease in nanoparticle size was observed across elution fractions, with fraction F3 consistently demonstrating enrichment in small EVs (<200 nm, as defined by ISEV guidelines). Based on the combined assessment of particle yield, size distribution, and co-isolated proteins, we select F3 for our downstream experiments.
    ***Note:*** For other cell sources or if using different columns, the void volume and EV-enriched fractions may differ. Therefore, initial characterization is recommended to define the optimal fractions for downstream use. Fraction selection should be tailored to the downstream application.
    ***Note:*** In our practice, EVs are used within 3 months of storage at −80 °C. Although some groups have reported stability with longer-term storage at −80 °C, a decline in particle concentration may occur over time. Supplementing PBS with human albumin and trehalose might improve EV stability and enables long-term storage, potentially for up to 2 years.^5^

## EV characterization

### Imaging flow cytometry

**Timing: 4–6 h (total)**

**Timing: 2 h (step 7)**

**Timing: 1–2 h, based on the number of EV samples (step 8)**

**Timing: 1–2 h, based on the number of EV samples (step 9)**

This step enables EV characterization at a single-vesicle level using *Amnis ImageStream*. It provides information about the abundance of EVs in the sample, their morphology, structural integrity, and surface marker expression. In our practice, we stain EVs first with carboxyfluorescein succinimidyl ester (CFSE) which selectively labels vesicles with intact lipid bilayers. This allows the gating of intact EVs for subsequent analysis. For surface marker profiling, we focus on the tetraspanins CD9, CD63, and CD81, which are commonly found on the surface of EVs. In our workflow, we typically perform single-marker APC staining on CFSE-labeled EVs. Double or triple staining remains an option depending on the experimental design, provided that fluorochromes with sufficiently distinct emission spectra are selected.

To proceed, prepare the following tubes, 30 μL each:

***Note:*** All antibodies used here (anti-CD63, anti-CD81, and anti-CD9) are Allophycocyanin (APC)-conjugated (*Exbio*). Fluorescence spillover between APC in these preparations and CFSE is negligible. However, fluorophore behavior may vary depending on the antibodies used. We therefore recommend assessing potential spillovers at least during the initial experiment. For double or triple antibody-staining experiments, the use of appropriate compensation controls is critical and should be performed for every run.

7. Sample preparation.
  a. Transfer the total volume of antibody required for the entire experiment (*see* ***Note*** *below*) into a labeled 1.5 mL Eppendorf tube and dilute in filtered PBS to achieve the desired concentration. Below are our working antibody concentrations (Table 4):
    ***Note:*** To calculate the total required antibody volume, multiply the number of tubes that will receive that antibody by 7.5, then adjust for the dilution factor.
    *Example:* if you need to add anti-CD63 to 3 EV tubes, then prepare 3 tubes × 7.5 μL/tube = 22.5 μL of anti-CD63 diluted in PBS, which corresponds to 2.25 μL of stock undiluted antibody.
    ***Note:*** PBS should be filtered with a 0.1 μm filter to remove particulates that could mimic the size of EVs and lead to false-positive events.
    **CRITICAL:** Antibody dilution is critical. Optimal final concentration depends on several factors, including cell type, EV concentration in the sample, and antibodies used. This protocol outlines the procedure for adipose and bone marrow-derived MSC-EVs prepared as described above, and using *ExBio* antibodies listed in this document. If alternative antibodies are used, titration is necessary to determine the most appropriate working concentration.
  b. Spin the diluted antibodies at 17,000 × *g* for 30 minutes at 4°C to remove aggregates and clumps. Transfer supernatants to new Eppendorf tubes and label them.
    **CRITICAL:** Pre-centrifugation of antibodies and dyes helps to remove aggregates. Some dyes or antibody-fluorophore conjugates can aggregate or form micelles that mimic the size and fluorescence of EVs, leading to false-positive events.^6^
  c. Prepare the samples in 1.5 mL Eppendorf tubes to achieve a target final volume of 30 μL, with an equal EV volume of 7.5 μL and antibody volume of 7.5 μL in each flow tube. Add PBS as needed to reach the target final volume of 30 μL (refer to Table 3).
    ***Note:*** The antibodies will be further diluted 4 times when added to the EV samples. If the initial dilution used is 1:10, the actual final dilution will be 1:40 (Table 4).
    **Pause point:** When staining samples, start by incubating EVs with diluted CFSE on ice in the dark for 15 min, then add diluted surface marker antibodies. Once all samples are prepared, incubate on ice in the dark for 1 h before acquisition.
8. Sample acquisition with *Amnis ImageStream.*
  a. Turn on the following channels, and put lasers at maximum voltage:
    i. Channels 1 and 9 for forward scatter (FSC) or Brightfield.
    ii. Channel 6 for side scatter (SSC).
    iii. Channel 2 for CFSE label: 488 nm laser at 200 mW voltage.
    iv. Channel 11 for APC stain: 642 nm laser at 150 mW voltage.
  b. Turn Gain mode ON, set magnification to 60×, and adjust Fluidics to low speed and high sensitivity. Open the Fluidics window from the Advanced tab.
  c. Create the following scatterplots:
    i. Area Ch01 (FSC/Brightfield) on x-axis vs. Intensity Ch06 (SSC) on y-axis.
    ii. Intensity Ch02 (CFSE fluorescence) on x-axis vs. Intensity Ch06 (SSC) on y-axis.
    iii. Intensity Ch11 (APC fluorescence) on x-axis vs. Intensity Ch06 (SSC) on y-axis.
      ***Note:*** These scatterplots can be saved as a template (*.ast*) for future experiments.
  d. Create a folder in which all files will be saved.
    ***Note:*** Acquired data will be saved as a raw image file (*.rif*).
  e. Press Load and enter the first negative control tube name in the Filename field. Vortex the tube, and when prompted, place it into the sample loader. Detected events will start appearing in the channels and on the scatterplots.
  f. Check the flow rate in the Fluidics window. Wait until the flow rate stabilizes before starting the acquisition. Once stable, begin acquisition and record events over a fixed period of time: 1 minute, in our practice.
    **CRITICAL:** Make sure the maximum total event counts is set high (>100,000). Otherwise, the acquisition may stop before the full 1-min run is completed.
    ***Note:*** Ensuring a stable sample flow rate helps maintain consistent sample delivery throughout acquisition. Fluctuations could result in inaccurate measurements.^7^
  g. Load the remaining negative control and experimental tubes one after the other (restart from step 2.e.) and acquire each sample over 1 minute.
  h. If indicated, run the compensation tubes after all experimental samples are acquired. Launch the compensation wizard. The same lasers will be selected, except for the scatter lasers, which turn off automatically for compensation. Follow the prompts and draw, on the scatterplots, the region that defines positive events. Plan to acquire 1,000 positive events. The compensation coefficients will be automatically calculated and can be saved to a compensation matrix file (*.ctm*).
9. Data analysis with IDEAS.
  a. Launch IDEAS (Image Data Exploration and Analysis Software). This software is used to visualize and analyze data generated by the imaging flow cytometer.
  b. Open sample raw file “EVs with CFSE in PBS” (*.rif*), select the template created in step 2c (*.ast*), and select the compensation matrix created in step 2h (*.ctm*).
    ***Note:*** If no compensation matrix is created (no spillover), this field can be kept empty.
    ***Note:*** File extensions:
    *.rif* = raw image file.
    *.cif* = compensated image file. It will be the same *.rif* file if no compensation.
    *.daf* = data analysis file. It contains all created plots, gates, regions, statistics, etc.
    Once the first *.daf* file is created, it can be used as a template to analyze the subsequent samples.
  c. Refer to Figure 3 for sequential gating strategy. Gate, on scatterplot ii, low-SSC and CFSE-positive events. Label this population as EVs. Apply this population to scatterplot iii. Gate, on scatterplot iii, the region defining APC positivity. The sample “EVs with CFSE in PBS” should be APC-negative.
    ***Rationale:*** EVs typically have a low SSC compared to cells or debris. Including only low scattering particles in the EV gate helps exclude large debris and aggregates (doublets/triplets). Additionally, EVs with intact lipid membranes are positive for CFSE. Gating out events that are CFSE-negative helps eliminate non-vesicular debris and disrupted EVs.
  d. Analyze the remaining experimental samples using the newly generated *.daf* as a template. View the Statistics table to identify EV subpopulations in each sample.
  e. Analyze negative control tubes to assess background noise. Background noise must be minimal to validate results. In our workflow, we accept a maximum of 10 false-positive events. If more than 10 false-positive events are detected, this should not exceed 5% of total events within the gated EV population.
    ***Optional:*** Use lysed EV controls to verify that CFSE signal and antibody staining disappear with membrane disruption.

**Table 1:** MSC culture environment for EV collection

Culture vessel	6-Well culture treated plate
Cells seeded per plate	0.5 × 10^6^ cells/plate
Seeding density	8.6 × 10^3^ cells/cm^2^
Serum-free xeno-free culture media	StemPro MSC complete
Media volume per well	2 mL
Conditioned media collection time points	Day 3 and Day 6
Total conditioned media volume for each EV preparation	12 mL (6 wells × 2 mL)

Culture conditions used for mesenchymal stromal cell expansion and EV collection under serum-free, xeno-free conditions.

**Table 2:** Composition of StemPro MSC complete serum-free xeno-free medium

MSC complete media components	Volume
StemPro MSC basal medium	49 mL
StemPro MSC supplement	0.5 mL
Glutamax (to be added at 100×)	0.5 mL
Gentamicin (stock 10 mg/mL, final 10 μg/mL)	50 uL
Total volume	5 mL

Volumes of each medium component used to prepare a total final volume of 50 mL of StemPro MSC complete serum-free xeno-free medium are shown.

**Table 3:** Flow cytometry controls and experimental tubes

Negative control tubes *(5 in total for each experiment)*	PBS alone (30 μL). PBS (22.5 μL) with CFSE (7.5 μL). PBS (22.5 μL) with anti-CD63 (7.5 μL). PBS (22.5 μL) with anti-CD81 (7.5 μL). PBS (22.5 μL) with anti-CD9 (7.5 μL).
Compensation tubes (*see Note*) *(4 in total for each experiment)*	Choose any EV sample for compensation tubes: EVs (7.5 μL) with CFSE (7.5 μL) in PBS (15 μL). EVs (7.5 μL) with anti-CD63 (7.5 μL) in PBS (15 μL). EVs (7.5 μL) with anti-CD81 (7.5 μL) in PBS (15 μL). EVs (7.5 μL) with anti-CD9 (7.5 μL) in PBS (15 μL).
Experimental EV tubes *(4 in total for each EV sample)*	Unstained EVs (7.5 μL) in PBS (22.5 μL). EVs (7.5 μL) with CFSE (7.5 μL) and anti-CD63 (7.5 μL) in PBS (15 μL). EVs (7.5 μL) with CFSE (7.5 μL) and anti-CD81 (7.5 μL) in PBS (15 μL). EVs (7.5 μL) with CFSE (7.5 μL) and anti-CD9 (7.5 μL) in PBS (15 μL).

Description of experimental and control tubes used for EV imaging flow analysis, including staining and negative control conditions.

**Table 4:** Working concentrations for imaging flow cytometry

Antibody or label	Initial dilution in PBS	Final dilution in EV/control sample
Anti-CD63 APC (*ExBio*)	1:10	1:40
Anti-CD81 APC (*ExBio*)	1:10	1:40
Anti-CD9 APC (*ExBio*)	1:10	1:40
CFSE (*Invitrogen*)	1:250	1:1000

Final working concentrations of antibodies used for imaging flow cytometry-based EV characterization.

**Figure 2:**
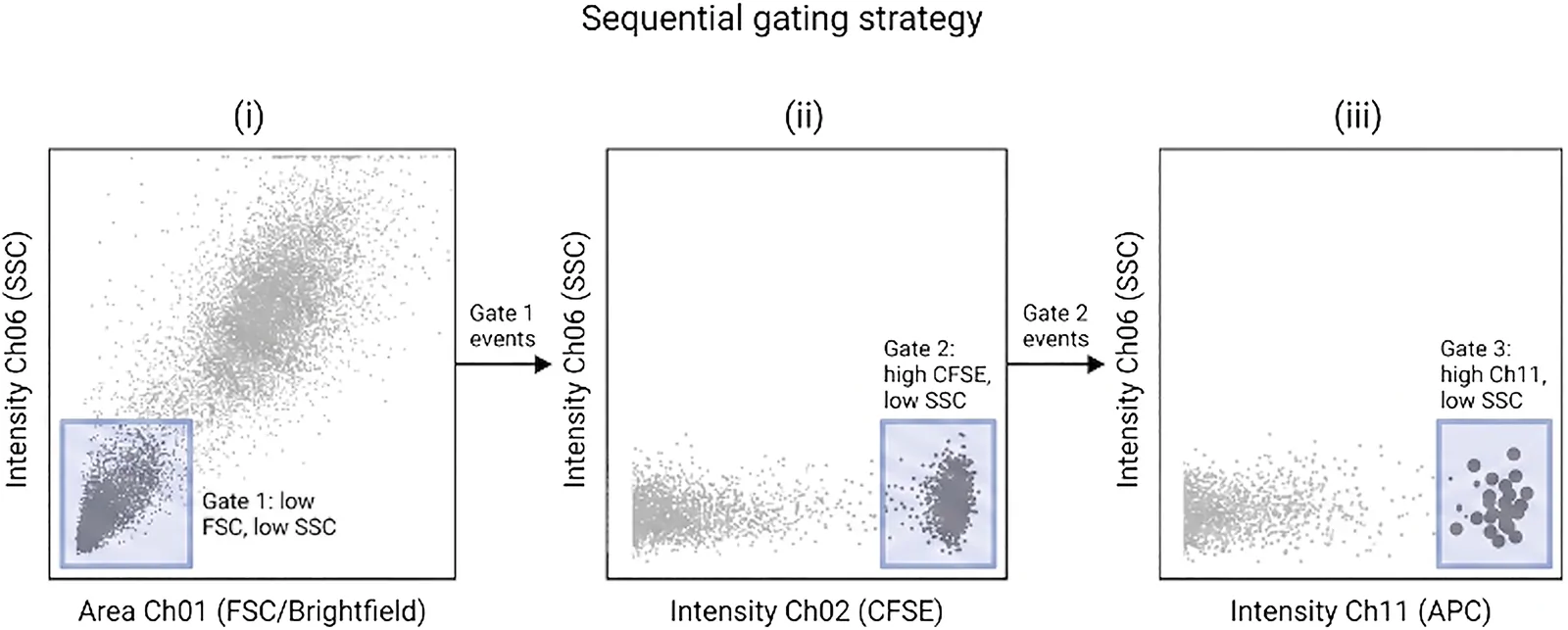
Sequential gating strategy for MSC-EV flow cytometry analysis EVs are first gated based on low forward scatter area (FSC-A) and side scatter area (SSC-A) properties to exclude cells, larger debris and aggregates. Next, CFSE-negative events are gated out to eliminate non-vesicular small debris and disrupted EVs. Antibody fluorescence is then used to characterize EV subpopulations based on surface marker expression. *Created with BioRender*.

**Figure 3:**
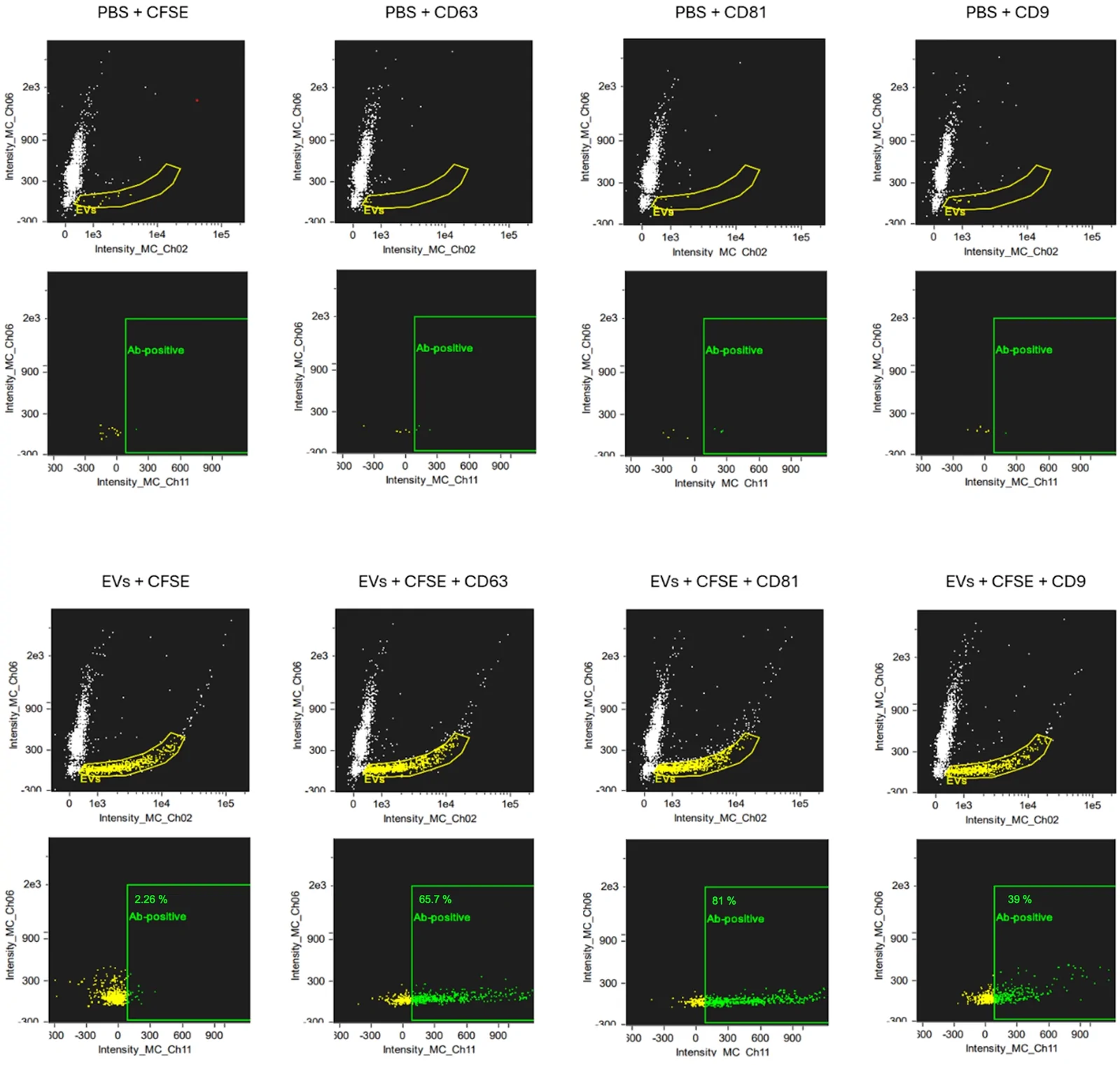
Flow cytometry characterization of MSC-derived EVs Imaging flow cytometry of CFSE-positive EVs shows robust expression of canonical tetraspanin markers (CD63, CD81, CD9) with minimal background in negative controls.

### Transmission electron microscopy

**Timing: 2 h (total)**

**Timing: 30 min (step 10)**

**Timing: 1 h 30 min (step 11)**

Transmission Electron Microscopy (TEM) allows us to visualize individual EVs and assess their morphology, size, and integrity. It helps distinguish EVs from non-vesicular contaminants such as protein aggregates and lipoproteins. EVs typically appear as round or oval structures, with the commonly observed cup-shaped morphology largely attributed to dehydration during negative staining rather than their actual structure. While TEM provides qualitative information on EV size heterogeneity and sample purity, it does not quantify particle concentration and is therefore best used in combination with other characterization techniques such as nanoparticle tracking analysis and imaging flow cytometry.

While we focus here on negative staining, we acknowledge other positive-labeling approaches such as immunogold staining, which can be used to confirm the presence of specific EV surface markers and further support vesicle identity.

10. Sample preparation (total of 3 EV samples).
  a. Prepare a solution of distilled water (DW) and 1% Uranyl Acetate (UA): Place a piece of parafilm in a clean petri dish. On the parafilm, dispense 3 drops of DW and 2 drops of 1% UA for each EV sample. Label the grid as below (Table 5).
  b. Using precision straight tweezers, transfer 3 carbon film-supported TEM copper grids onto a parafilm-covered slide, with the dark (carbon) side facing up.
  c. Follow the prompts on Denton Vacuum (or a similar film deposition instrument) to coat the grid surfaces. This sputtering step takes 1 minute and promotes the adhesion of EV samples to the grids.
  d. Using a straight cross-locking tweezer, hold one sputtered grid with the carbon-coated (dark) side facing up.
  e. Transfer 5 μL of the EV sample onto the carbon-coated side of the grid. Wait 1 minute, then gently blot off excess liquid using filter paper.
  f. Wash the grid by sequentially dipping it, carbon side down, into the 3 prepared droplets of DW, blotting gently with filter paper after each dip.
  g. Stain the grid by dipping it, carbon side down, into the 2 prepared droplets of UA, leaving it for 30 seconds on each droplet. Blot off excess liquid, then transfer the grid to a new labeled parafilm-covered petri dish.
  h. Repeat steps e-g for the remaining EV samples. Allow the grids to air-dry for a few minutes before imaging.
11. Imaging with Hitachi HT-7700 transmission electron microscope.
  a. Load the dried grids into the microscope specimen holder and carefully insert the holder into the column. Evacuate the column (switch to Evac). When the green lamp turns on, advance the holder fully into position.
  b. Launch AMT Image Capture Engine and enable auto HV (Horizontal/Vertical image orientation). When accelerating voltage reaches 80 kV, turn on Filament Current and Beam to initiate electron emission.
  c. Use “Stage Control” to visualize specimens one by one. Specimen number 1 corresponds to the most distal grid on the holder.
  d. Begin live imaging (Camera in, Quality Live on). Survey the grid at low magnification to identify regions with even stain and minimal contamination.
  e. Switch to higher magnifications to visualize individual EVs. Adjust focus (with the help of the wobbler function, WOB), beam intensity (brightness), and astigmatism (X and Y knobs) to optimize contrast while minimizing beam damage.
  f. Create a case study for each sample (File > Create case study). Acquire and save EV images. Include measurements.
  g. Return to low magnification when moving from one specimen to another. Continue imaging until all desired data is captured.

***Note:*** Refer to the user manual of AMT Image Capture Engine for further details if needed. The manual is available at: https://0201.nccdn.net/4_2/000/000/038/2d3/AMT-Camera-Manual.pdf.

**Table 5:** Grid preparation for negative staining prior to TEM

	EV sample 1	EV sample 2	EV sample 3
DW	o	o	o
DW	o	o	o
DW	o	o	o
UA	o	o	o
UA	o	o	o

Drops of distilled water (DW) and 1% uranyl acetate (UA) applied over a parafilm in preparation for transmission electron microscopy.

**Table 6:** UPLC gradient parameters for LC–MS/MS analysis

Time	Flow (μL/min)	%B
0.0	0.3	4
10.0	0.3	4
50.0	0.3	30
60.0	0.3	90
70.0	0.3	90
72.0	0.3	4
90.0	0.3	4

Gradient parameters for the reversed phase UPLC separation used to separate peptides for elution directly into the Orbitrap Eclipse Tribrid mass spectrometer. All solvent ramp events are linear.

**Table 7:** Mass spectrometry acquisition parameters

	Parameter	Setting
**MS1 acquisition**	Resolution	120,000
	AGC target	4 × 10^5^
	Maximum ion time	Auto
	RF lens	30%
	Scan range	300–1800 m/z
**MS2 acquisition**	Acquisition mode	Data-dependent
	Resolution	15,000
	Intensity threshold	> 5.0 × 10^4^
	AGC target	Standard
	Maximum ion time	Auto
	Mass range	Normal
	Scan range	Auto
	Isolation window	1.6 m/z
	Cycle time	Fixed at 3 seconds
	Fragmentation	HCD, 30% energy
	Charge states	2–8

Mass spectrometry acquisition parameters used for proteomic analysis of EV samples.

### Nanoparticle tracking analysis

**Timing: 2 h**

Nanoparticle tracking analysis (NTA) is a light-scattering-based technique used to characterize EVs by tracking their Brownian motion in suspension. We analyze our samples using ZetaView platform (*Particle Metrix*). Although the system can also perform fluorescence-based measurements, in our workflow it is primarily used to determine EV size distribution and particle concentration. In addition. zeta potential, which reflects particle stability in suspension, can be measured.

The main steps of the NTA workflow using the ZetaView platform are:

12. Instrument setup and calibration: Run the auto-setup wizard and follow prompts to rinse the measurement cell. Load 100 nm polystyrene calibration beads (supplied with the system) at 1:250,000 dilution and press Start. This step calibrates the system and ensures accurate zeta potential measurement.
13. Sample preparation and dilution: Dilute EV samples in filtered PBS to achieve optimal particle concentration. Start first with diluting to 1:300, then dilute further if particle counts exceed 350 particles per frame during acquisition.

***Note:*** The dilution factor should be adjusted to obtain approximately 100 to 200 detected particles per frame, which is considered optimal for ZetaView analysis, although up to 350 particles per frame may still be acceptable. Samples prepared as described above are typically diluted 300× to 1000×.

14. Sample loading: Load diluted samples into the measurement cell using a syringe while ensuring that no air bubbles are introduced. Flush the system with filtered PBS between samples to minimize carryover.
15. Data acquisition: Perform measurements at 25°C with the 488 nm laser on. For each sample, videos are acquired across 11 distinct positions within the measurement cell while particles undergo Brownian motion.
16. Data analysis: The software tracks individual particles to calculate size distribution, particle concentration and zeta potential.

***Note:*** To ensure accuracy, perform all measurements in replicates under identical acquisition conditions.

***Note:*** Particle Metrix provides valuable online resources, including webinars and instructional videos, that may assist users with the NTA workflow and ZetaView operation. These resources are available at: https://www.particle-metrix.com/pages/webinars-videos.

### EV-protein extraction and digestion

**Timing: ∼2–8 h (step 17)**

**Timing: 2.5 h (step 18)**

**Timing: Overnight (step 19)**

**Timing: ∼1.5 h (step 20)**

**Timing: 0.5 h (step 21)**

**Timing: 90 min/sample (step 22)**

Bottom-up proteomic analysis utilizing liquid chromatography and high-resolution mass spectrometry (LCMS) allows robust identification of thousands of proteins from a single analysis, with an associated quantitative value over a dynamic range of 4–5 orders of magnitude. This technique provides an in-depth characterization of the protein content of EVs.

***Note:*** The following protocol can be done either at a proteomics mass spectrometry (MS) facility or a research lab with mass spectrometry instrumentation, or the workflow can be split between the research lab and an MS facility. If the research lab chooses to perform just the first part of the protocol, samples can be submitted to a facility after step 1, 2, 3, or 4. LCMS-grade solvents will be required starting at step 4.

17. EV concentration and lysis.
  a. Make a 1 L 100 mM ammonium bicarbonate solution (pH 8) in LCMS grade water (7.91 g/L) for use in subsequent steps.
  b. The 700 μL volume from F3 of the isolated samples should be evaporated to allow for change of buffer with minimal loss of sample. Eppendorf tubes containing the samples should be dried to completion in a speedvac concentrator. Lyophilization may be used as an alternative. This step will take several hours to complete, but the exact timing depends on the strength of the vacuum, the temperature of the centrifuge chamber, and the composition and total volume of solvent being evaporated from all samples being dried.
    ***Note:*** The speedvac used at our Proteomic Core facility is a LabConco Acid-Resistant CentriVap concentrator combined with a CentriVap −50°C cold trap. The centrifuge portion is heated to 40°C during evaporation. No other programming is applied. The samples are monitored manually until evaporation is complete.
    ***Note:*** We do not recommend an additional buffer exchange step at this point in the protocol, as no evidence of trypsin inhibition was observed, and additional column- or filter-based processing may incur further sample loss from adsorption. EV samples have limited starting material and minimizing any possible source of loss during sample preparation is critical to maintain adequate sampling depth.
  c. Resuspend the dried samples in 20 μL total volume of 50:50 (v/v) trifluoroethanol (TFE) and 100 mM ammonium bicarbonate and mix gently by pipetting. The addition of TFE lyses the EV capsids and allows access to the internal contents, while ammonium bicarbonate buffers the pH of the protein environment for subsequent preparation steps.
18. Sample reduction and alkylation.
  a. Just before use, make a 1 mL solution of 100 mM dithiothreitol (DTT) (15.4 mg/mL) in 100 mM ammonium bicarbonate. Premade frozen stocks of DTT are less effective due to oxidation rates.
  b. Add 1 μL of DTT stock to samples for a final concentration of 5 mM (20× dilution). This step reduces any disulfide bonds between cysteine residues.
  c. Incubate at 25°C for 60–90 min on a thermal mixer with constant shaking at 800 rpm.
  d. Just before use, make a 1 mL solution of 100 mM iodoacetamide stock (18.5 mg/mL) in 100 mM ammonium bicarbonate.
    ***Note:*** Iodoacetamide undergoes UV-induced degradation on the order of 70% loss in 20 min. This reagent should be made directly before use and must be protected from light during the reaction step.
  e. Add 2.1 μL iodoacetamide stock to samples for a final concentration of 10 mM (10× dilution). This step chemically modifies the reduced cysteine residues with an alkyl group, so that disulfide bonds cannot be re-formed. Iodoacetamide is used at 2× the concentration of DTT to account for side reactions with unused reagent from the last step.
  f. Protect from light and incubate at 25°C for 45 min on a thermal mixer with constant shaking at 800 rpm.
  g. Add 207 μL of 100 mM ammonium bicarbonate for a total sample volume of 230 μL. This step is to dilute the TFE such that it will not interfere with the structure and inhibit activity of trypsin to be added.
19. Protein proteolysis.
  a. If a protein quantification assay was performed (microBCA assay, UV-Vis absorbance at 280 nm, or estimation of band density using sodium dodecyl sulfate-polyacrylamide gel electrophoresis (SDS-PAGE)), the total amount of trypsin to add should be calculated based on a 1:20 (w/w) enzyme:sample ratio.
  b. Thaw a vial of porcine modified sequencing grade trypsin. This is supplied by the vendor as a frozen liquid stock at 0.4 mg/mL, at low pH which inhibits the enzyme. Protease activity will be restored upon dilution into the basic pH of the sample.
    ***Note:*** Trypsin activity decreases with successive freeze-thaw cycles. We do not recommend using aliquots past the second thawing.
  c. Add trypsin at a 1:20 (w/w) ratio of enzyme:total sample protein. Check a trace amount of sample on a pH strip after trypsin addition to confirm the solution is between pH 7–8.5. If it is acidic, adjust as needed. Be sure the Eppendorf lid is firmly closed.
  d. Incubate overnight (12–16 hrs) at 37°C on a thermal mixer with constant shaking at 800 rpm.
20. Peptide desalting.
  a. Quench the protease reaction by acidifying with LCMS grade neat formic acid to pH < 3 (usually requires an acid volume of ∼1 to 2% sample volume). Confirm pH by testing a trace amount on a pH strip.
  b. Add 100% acetonitrile to a final concentration of 5%. If 2 μL of formic acid was sufficient to adjust the pH in the previous step, the total volume now should be ∼233 μL, so add 12.2 μL acetonitrile.
    ***Note:*** Solvents used for desalting include water, acetonitrile, and formic acid. From this step forward, all solvents used should be Fisher Optima LCMS grade. Milli-Q water or HPLC water is not sufficiently pure. If you do not have LCMS grade solvents in lab, your institution’s mass spectrometry facility will likely have aliquots available to you.
  c. Desalt samples using Pierce C18 spin columns, following the instructions provided by the manufacturer with two exceptions: we recommend using formic acid in place of trifluoroacetic acid and using acetonitrile in place of methanol.
  d. Dry the desalted elutions to completion in a speedvac concentrator, as described in Step 1b of this section.
21. Peptide resuspension, quantification, and normalization.
  a. Once samples are completely dry, tubes should appear empty.
  b. Resuspend samples in 12 μL of Solvent A (Fisher Optima LCMS water + 0.1% formic acid), by pipetting.
  c. Use 1 to 2 μL to measure the total peptide concentration by NanoDrop, using the protein quantification function and Solvent A as the blank. The default assumption of 1 absorbance unit = 1 mg/mL is appropriate here, as the protein composition and true extinction coefficient of the sample is unknown.
  d. Based on the lowest measured concentration, calculate the dilutions needed for a 10 μL volume of each sample at the same concentration. This will help ensure that protein quantitative differences across samples reflect biological changes and not simply different injection amounts.
  e. Aliquot the calculated amount of sample and LCMS-grade Solvent A into injection vials labeled with sample name, concentration, and date. Mix by gentle pipetting or by flicking. Do not create air bubbles.
22. LCMS acquisition.
  EV proteomes are considered a low-complexity sample compared to full cell lysates and typically do not need extended chromatographic separation for full characterization. The following parameters describe analysis using a 1-hour gradient LC separation with elutions being directly ionized into a Thermo Scientific Orbitrap Eclipse Tribrid mass spectrometer. Peptide analysis is performed using a Thermo Scientific Ultimate 3000 RSLCnano ultra-high performance liquid chromatography (UPLC) system coupled to a high-resolution Thermo Scientific Orbitrap Eclipse Tribrid mass spectrometer.
  a. Inject each sample onto a nanoEase M/Z Peptide BEH C18 column (1.7 μm, 75 μm × 250 mm, Waters Corporation) heated to 50°C, and separated by reversed-phase UPLC using a gradient of 4%–30% Solvent B (0.1% formic acid in Fisher Optima LC/MS grade acetonitrile). Table 6 describes the LC gradient parameters:
  b. Elute peptides directly into the Eclipse using positive mode nanoflow electrospray ionization with a capillary voltage of 2200 V. Acquire MS1 scans and data-dependent MS2 scans as described in Table 7. Set dynamic exclusion to exclude for 30 sec after 1 observation, for charge states 2–8 only.

**Table 8:** MaxQuant search parameters

Parameter	Setting
Software	MaxQuant (v2.0.2.0)
Search engine	Andromeda
Database	Human UniProt proteome (UP000005640, downloaded Jan 2, 2025) and MaxQuant contaminants database
Protease specificity	Trypsin/P
Missed cleavages	Up to 2
Minimum peptide length	5 residues
Maximum peptide mass	4600 Da
Variable modifications	Up to 5 variable modifications per peptide are considered from the following list: Met oxidation, protein N-terminal acetylation, Asn/Gln deamidation, and Gln conversion to pyro-Glu
Fixed modifications	Cysteine carbamidomethylation

MaxQuant database search and protein identification parameters used for EV proteomic data analysis.
23. Peptide and protein identification and quantification.
  Raw DDA MS files may be searched with a variety of commercial or non-commercial softwares. We recommend using MaxQuant, which is freely available for use on Windows platforms. The speed of the search step will depend strongly on the number of raw files and the number of possible peptide forms included in the search. The following parameters describe analysis of 5 raw files using MaxQuant on a high performance computing cluster, and took about 7 hours to complete.
  a. Identify peptides using MaxQuant software (v2.0.2.0) and its embedded Andromeda search engine, and quantified using label-free quantification.^8^ Search and identification parameters are summarized in Table 8.
  b. Filter results to a 1% false discovery rate at the peptide and protein levels using the target-decoy approach; all other parameters are left with default values.
  c. Perform subsequent data visualization and analysis with Scaffold (v5, Proteome Software, Inc.) and msDiaLogue (Xiao S, Moore T, Watt C (2025). *msDiaLogue: Analysis and Visuals for Data-Independent Acquisition Mass Spectrometry Data.* R package version 0.0.6, https://github.com/uconn-scs/msDiaLogue).

### EV-RNA extraction and quality assessment

**Timing: 2 h**

The following steps were adapted from Qiagen exoRNeasy Midi/Maxi Kit manual, with modifications to optimize RNA isolation from MSC-derived EVs. Due to relatively low EV-RNA yield, four MSC-EV samples (700 μL F3 each) are pooled for each EV-RNA preparation.

Although EVs were already extracted from conditioned media with SEC, we perform here an additional membrane-based EV affinity binding step to further remove any residual contaminants and ensure that the recovered RNA is exclusively vesicular.

24. Membrane-based EV affinity binding.
  a. For each EV-RNA preparation, combine, in a 15 mL tube, four F3 MSC-EV samples (700 μL each), derived from two MSC 6-well culture plates (Plate 1 and Plate 2) and collected at two time points (Day 3 and Day 6) (Table 9).
    ***Note:*** If EVs were frozen prior to RNA extraction, remove EV samples from – 80°C storage the day before isolation and thaw overnight at 4°C.
    **CRITICAL:** For RNA extraction, pool EVs from four independent EV isolations, rather than combining four conditioned media samples before EV isolation.
  b. Add one volume of Buffer XBP (2.8 mL of Buffer XBP to 2.8 mL of pooled EV samples). Mix gently.
  c. Load the sample/Buffer XBP mix onto a Maxi exoEasy spin column and centrifuge at 500 × *g* for 1 minute, followed by 3,000 × *g* for 1 minute. Discard the flow-through.
  d. Add 10 mL Buffer XWP to the column, and centrifuge at 3,000 × *g* for 5 minutes. Discard both the flow-through and the collection tube. Place the spin column into a new Maxi collection tube.
  e. Add 700 μL QIAzol directly to the column membrane and centrifuge at 3,000 × *g* for 5 minutes. Transfer the resulting lysate to a new collection tube.
25. EV-RNA isolation.
  a. Vortex the lysate briefly and incubate at room temperature for 5 minutes.
  b. Add to the lysate 90 μL of chloroform, shake for 15 seconds, and incubate at room temperature for 2 minutes.
  c. Centrifuge at 12,000 × *g* for 15 minutes at 4°C to achieve phase separation.
  d. Carefully transfer the upper aqueous phase to a new tube and add 2 volumes of 100% ethanol, mixing gently.
  e. Load 700 μL of the sample onto an RNeasy MinElute spin column (pre-cooled at 4°C) and spin at 8,000 × *g* at room temperature for 30 seconds. Discard the flow-through and repeat until the entire sample has been processed.
  f. Add 700 μL of Buffer RWT to the column and spin at 8,000 × *g* at room temperature for 30 seconds. Discard the flow-through.
  g. Add 500 μL of Buffer RPE to the column and spin at 8,000 × *g* at room temperature for 30 s. Discard the flow-through.
  h. Add an additional 500 μL of Buffer RPE to the column and spin at 8,000 × *g* at room temperature for 2 minutes. Discard both the flow-through and the collection tube.
  i. Place the column into a new collection tube and spin at 16,000 × *g* at room temperature for 5 min to remove any residual buffer. Discard both the flow-through and collection tube.
  j. Transfer the column to a new collection tube and add 14 μL RNase-free water to the center part of the membrane. Let sit for 1 minute, then spin at 16,000 × *g* at room temperature for 1 minute to elute the RNA.
    ***Note:*** The recovered RNA volume is 12 μL, since about 2 μL is retained in the column.
  k. Immediately store the eluted RNA at −80°C.
26. EV-RNA quality assessment.

**Table 9:** EV samples combined for each EV-RNA preparation

Plate	Collection day (D)	Sample name
Plate 1	Day 3	Plate 1 F3 D3
Plate 1	Day 6	Plate 1 F3 D6
Plate 2	Day 3	Plate 2 F3 D3
Plate 2	Day 6	Plate 2 F3 D6

F3 refers to the third fraction collected during size-exclusion chromatography. D refers to the day of collection of conditioned media.

EV samples combined for each RNA extraction. F3 indicates the third size-exclusion chromatography fraction, and D indicates the day of conditioned media collection.

Assess RNA size distribution and quantity using High Sensitivity RNA ScreenTape assay (Agilent Tapestation).

***Note:*** The High Sensitivity RNA ScreenTape assay works well for low-input EV-RNA profiling. For further quality assessment focused on small RNA in the sample, the use of Agilent Bioanalyzer small RNA kits is strongly encouraged.

***Note:*** EV-RNA is naturally depleted of intact ribosomal RNA and is instead enriched in small RNAs with short nucleotide sequence lengths. As a result, RNA integrity number (RIN), typically used to evaluate the quality of cellular RNA, is not applicable for EV-RNA quality assessment.

***Note:*** In our experience and as reported by other groups, electrophoretic platforms (Agilent Tapestation and Bioanalyzer systems) are more suitable for EV-RNA quality assessment than spectrophotometric or fluorometric methods (NanoDrop and Qubit). This is likely due to the very low abundance and atypical composition of RNA within EVs.^9^

## Expected outcomes

Application of this protocol is expected to yield a reproducible and well-characterized population of MSC-derived EVs with phenotypic, structural, proteomic, and transcriptomic features, as described below.

Phenotypic characterization by imaging flow cytometry is anticipated to demonstrate robust expression of canonical tetraspanin markers, with approximately 60%–70% CD63-positive, 75%–85% CD81-positive, and 30%–40% CD9-positive vesicles. This analysis is performed on intact EVs identified as CFSE-positive events. Importantly, minimal to no background signal should be observed in negative controls when pre-centrifuged diluted antibodies and filtered PBS are used, supporting the specificity of the staining and the effectiveness of the filtering and cleanup steps (Figure 2).

**Figure 4:**
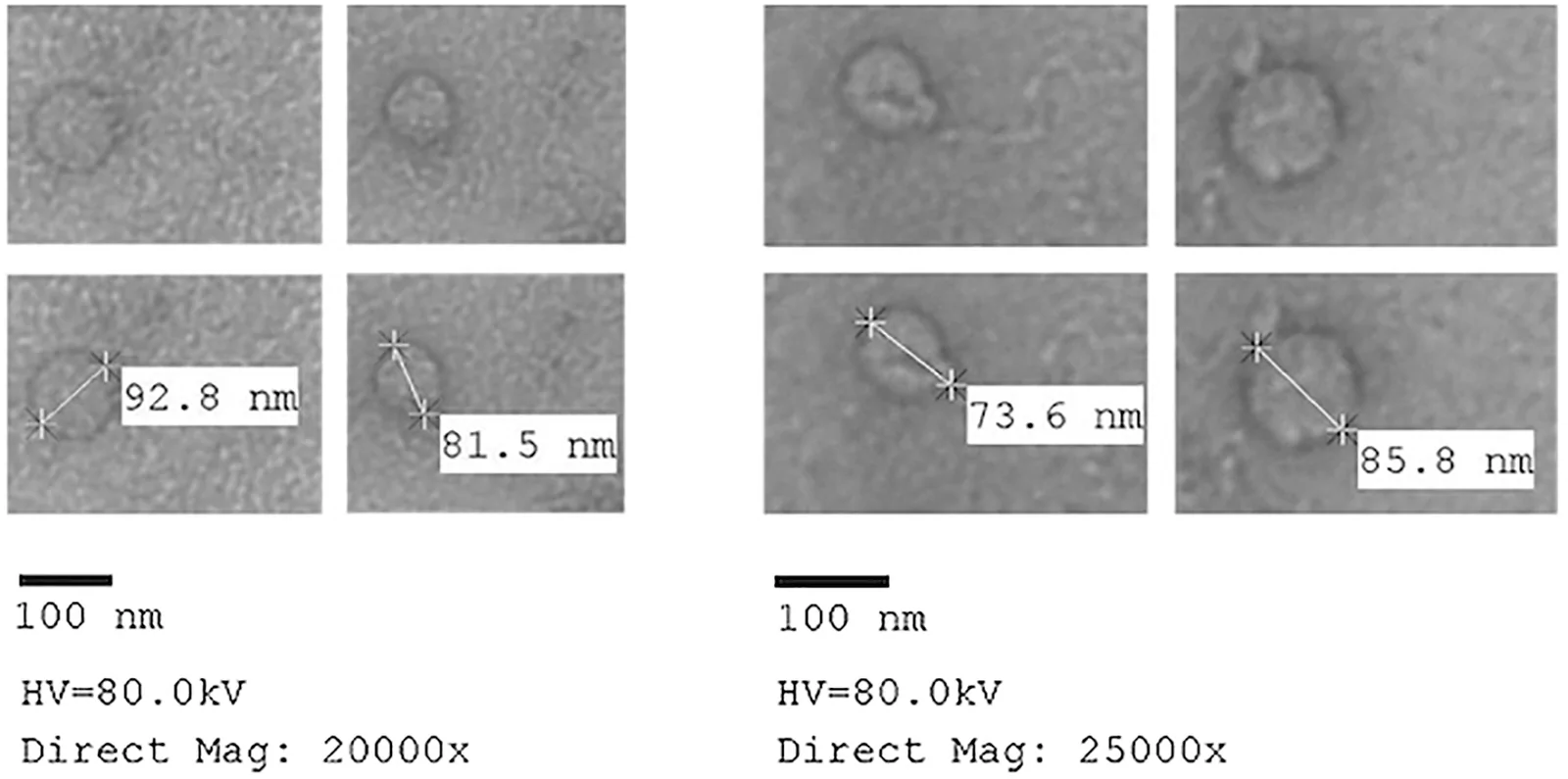
Morphological characterization of EVs by transmission electron microscopy Transmission electron microscopy shows EVs with round, oval, and cup-shaped morphologies.

TEM is expected to reveal vesicles with round, oval, or occasional cup- or donut-shaped morphologies. Particle sizes can range from 50 nm to 200 nm (Figure 4). As commonly observed, the apparent size assessed by TEM does not directly correlate with size distributions obtained by nanoparticle tracking analysis (NTA), reflecting differences between imaging-based and light-scattering–based measurement principles.

**Figure 5:**
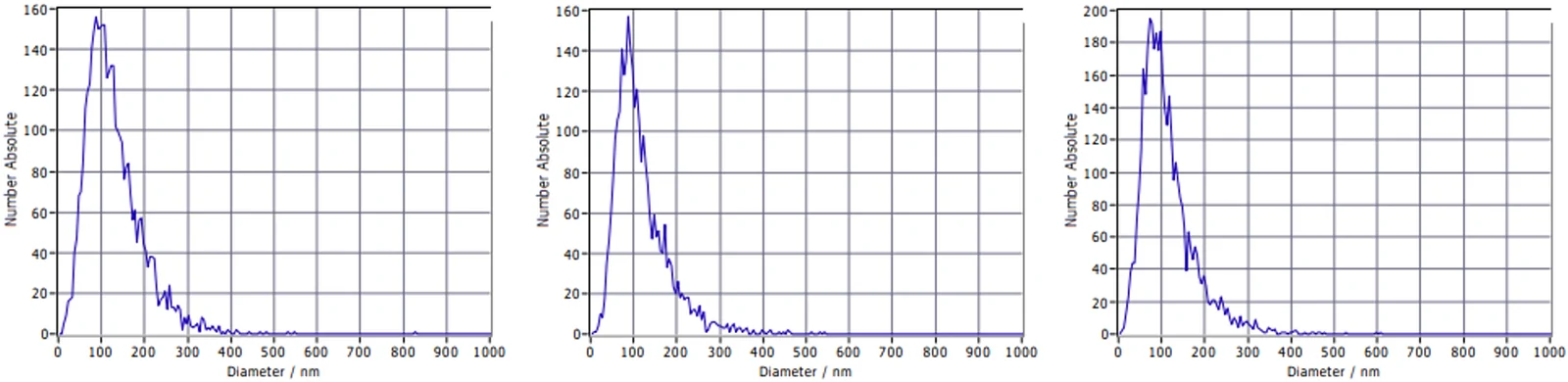
Size distribution of EVs on nanoparticle tracking analysis NTA demonstrates a particle size distribution enriched in small vesicles (mean diameter ∼100–160 nm) and a particle concentration on the order of 10^10^ particles/μL. Three EV samples results are shown.

NTA is routinely incorporated into our characterization strategy as it provides quantitative particle concentration and size distribution information that complements structural and phenotypic analyses. NTA performed on EV samples isolated using this protocol is anticipated to reveal a particle concentration on the order of 10^10^ particles/μL and a median particle diameter of approximately 100–180 nm, indicating that the EV population is enriched in small vesicles (Figure 5).

When samples are prepared as described in this protocol, the concentration of co-isolated soluble proteins in the retained fraction F3 is less than 60 ng/mL by BCA assay. On LC-MS, the major contaminant, albumin, accounts for no more than 10% of total spectra. Although contaminant abundance is low, their detection is consistent with the recognized challenge of achieving complete elimination of co-isolated non-EV proteins in EV preparations, even under optimized workflows. Despite serum-free xeno-free culture conditions, low-level background signals may still arise during EV processing or handling, including adsorption of trace proteins to plasticware, carryover from reagents, or incomplete removal of soluble proteins during isolation steps.

**Figure 6:**
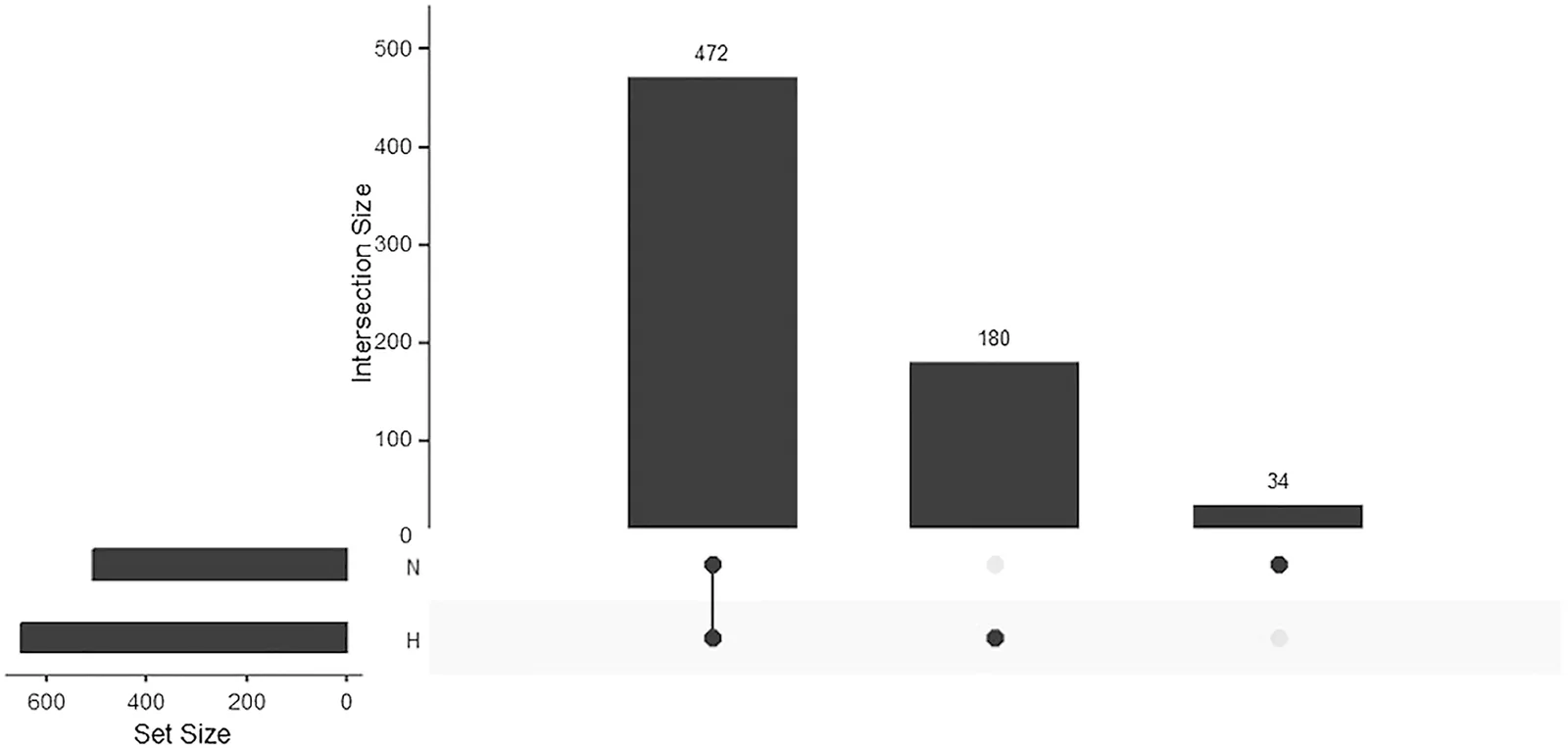
Upset plot of identified proteins within MSC-EVs from an experiment comparing two culture conditions (N refers to normoxic conditions 21%O_2_, H refers to hypoxic conditions 5%O_2_, with 2 and 3 replicates respectively) Data shown were searched against both the *Homo sapiens* reference proteome downloaded from UniProt and a database of common contaminant proteins and were filtered for proteins identified by at least 2 unique peptides. Proteins whose identity cannot be resolved from other isoforms or similar sequences on the basis of the peptides observed are clustered as a group. Of 686 total protein groups, 69% are found in both groups.

**Figure 7:**
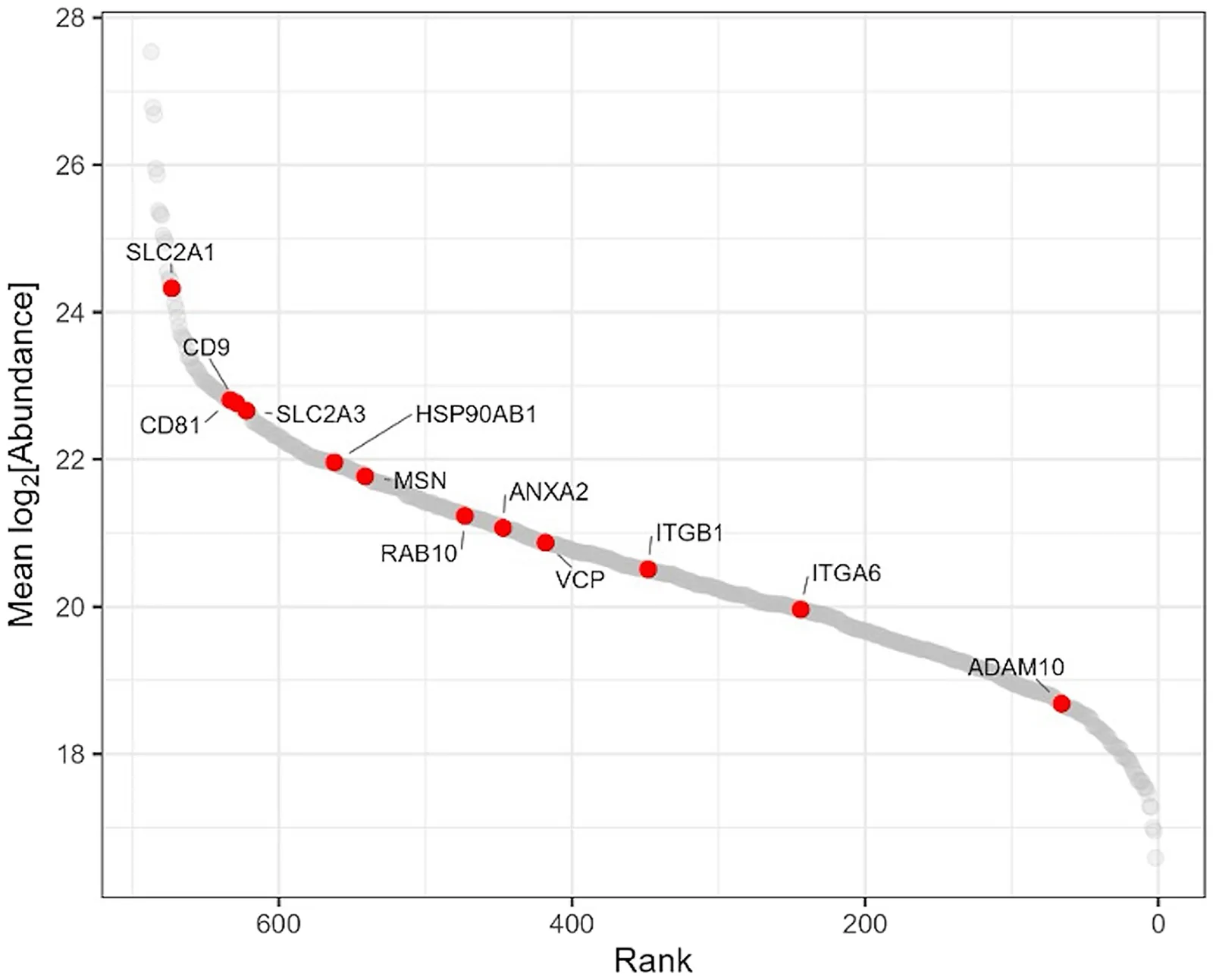
Whittaker plot of identified proteins, ranked by log2-transformed quantitative abundance averaged across all samples Selected EV-specific proteins are highlighted in red.

The depth of EV proteome characterization by LCMS depends on the amount of starting material, whether there are any dominating high abundance proteins that may suppress signals for other proteins (e.g., if the sample was isolated from plasma and contaminating plasma proteins such as albumin, immunoglobulins, or lipoproteins are present; or if the amount of protease used was in large excess), as well as the scan speed of the mass spectrometer and the acquisition type used. For data acquired on a high-resolution accurate-mass instrument with relatively fast scan speeds, the number of protein groups identified is expected to range between 300–800 (Figure 6). Specificity for EV content can be evaluated based on the detection of canonical EV proteins, such as heat-shock proteins, annexins, integrins (ITGA1, ITGA6, and ITGAB1), and CD-family proteins such as CD44, CD47, CD9, CD63, and CD81, as shown in Figure 7.^10^

**Figure 8:**
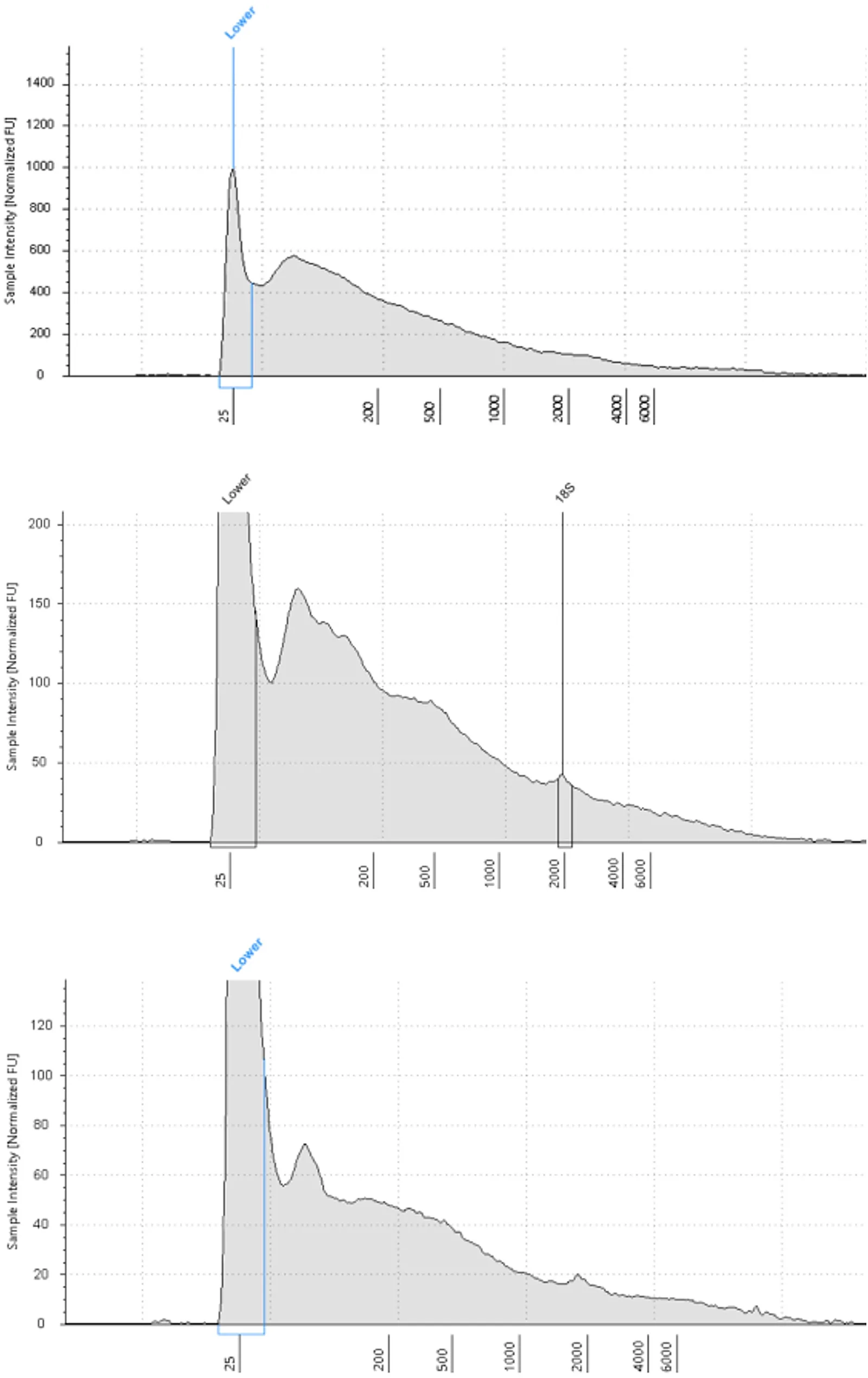
Size distribution of EV-associated RNA Agilent High Sensitivity TapeStation analysis shows EV-RNA predominantly within the small RNA range (<200 nt), with minimal ribosomal RNA peaks. Three EV samples results are shown.

RNA quality assessment using Agilent High Sensitivity TapeStation assay is expected to show that the majority of EV-associated RNA falls within the small RNA size range (<200 nt), with minimal or undetectable ribosomal RNA peaks (Figure 8). When the protocol is followed as described, including pooling at least four EV preparations for each RNA extraction procedure, it is expected to obtain 200–400 ng/μL of RNA in each sample. The marked predominance of small RNA is consistently observed across preparations despite the absence of any small RNA-specific enrichment steps during RNA extraction. This pattern reflects the natural enrichment of small RNAs within MSC-derived EVs rather than a technical artifact. The minimal presence of ribosomal RNA peaks further supports the purity of EV-associated RNA samples and suggests limited cellular contamination. These results validate that the protocol preserves endogenous EV RNA cargo and generates material suitable for downstream small RNA analyses.

## Limitations

This protocol is optimized for the isolation and characterization of EVs derived from MSCs cultured under serum-free xeno-free conditions. While it yields reproducible and well-characterized EV preparations, several limitations should be recognized.

First, the biological properties of the parental MSCs can influence EV yield and molecular composition. Parameters such as MSC source, donor variability, passage number, and metabolic state can all affect EV secretion and molecular content. This protocol was developed and validated using bone marrow- and adipose-derived MSCs from both cell lines and patient-derived samples at passages P2-P5. Nevertheless, differences in donor material or tissue origin may affect EV secretion rates and molecular content.

Second, from a technical perspective, although the combination of ultrafiltration and size-exclusion chromatography improves EV purity, complete selectivity is quite impossible, and co-isolation of soluble proteins may still occur. Accordingly, careful EV characterization and transparent reporting of the isolated population are always essential. Another practical limitation is the inherently low yield of EV-associated RNA, which often requires pooling of multiple EV preparations to obtain sufficient material for downstream analyses. While pooling improves RNA recovery, it limits the ability to assess biological variability between individual samples.

Finally, this protocol focuses on EV isolation and characterization and does not include functional assays. Any conclusion regarding EV biological potency should be validated using appropriate *in vitro* or *in vivo* functional studies.

## Troubleshooting

### Problem 1

Variable EV recovery or yield.

### Possible cause

Variable cell viability at time of seeding or inconsistent culture conditions.

### Potential solution

Confirm MSC viability exceeds 90% prior to seeding. Standardize seeding density, media volume, media composition, and conditioned media collection timepoints (Refer to Cell culture and collection of conditioned media). The conditions described in this protocol were optimized for adipose- and bone marrow-derived MSCs. Alternative cell sources may require re-optimization.

### Problem 2

High protein contamination.

### Possible cause

Residual proteins from culture media or supplements, or incomplete separation of EVs from soluble proteins.

### Potential solution

Use serum-free culture conditions. We suggest in this protocol the use of StemPro MSC Serum-Free Xeno-Free Medium (Refer to Cell culture and collection of conditioned media). As a negative control, process unconditioned media through the SEC column and measure protein content in collected fractions by BCA assay to assess background protein levels. Additionally, fraction selection may require optimization depending on the desired balance between EV yield and purity and the intended downstream application (Refer to EV isolation).

### Problem 3

Slow filtration through the Amicon Ultra-15 centrifugal filter unit.

### Possible cause

Membrane clogging due to excessive sample loading.

### Potential solution

Reduce the volume loaded onto each filter unit. Do not reload or reuse the same filter to process additional conditioned media (Refer to EV isolation). If concentrating larger volumes of conditioned media is desired, consider alternative filtration devices designed for higher capacities, such as the Centricon Plus-70 centrifugal filter (*Sigma-Aldrich*).

### Problem 4

High background signal or false-positive events during imaging flow cytometry.

### Possible cause

Unbound antibody, excess dye, or aggregates.

### Potential solution

Perform antibody titration to determine the optimal dilution for your specific EV preparation and antibody. Dilutions described in this protocol are optimized for *Invitrogen* CFSE dye and *ExBio* antibodies; alternative reagents may require re-optimization. Prior to staining, centrifuge antibodies and dyes to remove aggregates, then carefully transfer the supernatant without disturbing the pellet. Together, these steps reduce background noise and improve the specificity of EV detection (Refer to Imaging flow cytometry).

### Problem 5

Low EV-associated RNA yield.

### Possible cause

Insufficient starting EV material.

### Potential solution

EVs contain relatively low amounts of RNA; therefore, pooling at least four EV preparations (corresponding to approximately 48 mL of conditioned media) is critical at the RNA extraction step. Additional pooled EV preparations (or larger starting volumes of conditioned media) may be required depending on the desired RNA yield (Refer to EV RNA extraction and quality assessment).

## Resource availability

### Lead contact

Further information and requests for resources and reagents should be directed to and will be fulfilled by the lead contact, Hala Saneh (HSaneh@connecticutchildrens.org).

### Technical contact

Technical questions on executing this protocol should be directed to and will be answered by the technical contact, Abigail Collins (abcollins@uchc.edu).

### Materials availability

This study did not generate new unique reagents.

### Data and code availability

Relevant results are included within the article. Additional EV-RNA sequencing or proteomic results are available from the corresponding author upon reasonable request.

## Acknowledgments

The authors gratefully acknowledge the Connecticut Children’s Foundation and Dr. Catherine Stevenson and Dr. Keith Stevenson for supporting this work. The authors would also like to acknowledge the 10.13039/100000002NIH S10 High-End Instrumentation Award 1S10-OD028445-01A1, which supported this work by providing funds to acquire the Orbitrap Eclipse Tribrid mass spectrometer housed in the University of Connecticut Proteomics & Metabolomics Facility.

## Author contributions

A.C. and H.S. conceptualized the study and led the development of the overall protocol. A.C., J.W., H.W., and H.S. performed experiments and optimized extracellular vesicle isolation and characterization workflows. J.L. and J.B. led the development and optimization of the proteomics components of the protocol and provided expertise in mass spectrometry-based analyses. C.F. provided mentorship, scientific insight, and expertise. A.C., J.L., and H.S. wrote the manuscript with input from all authors. All authors reviewed, edited, and approved the final version of the manuscript.

## Declaration of interests

The authors declare no competing interests.
